# Extract of *Pinus densiflora* needles suppresses acute inflammation by regulating inflammatory mediators in RAW264.7 macrophages and mice

**DOI:** 10.1080/13880209.2022.2079679

**Published:** 2022-06-12

**Authors:** Seul-Yong Jeong, Won Seok Choi, Oh Seong Kwon, Jong Seok Lee, Su Young Son, Choong Hwan Lee, Sarah Lee, Jin Yong Song, Yeon Jin Lee, Ji-Yun Lee

**Affiliations:** a College of Pharmacy, Chung-Ang University, Seoul, Republic of Korea; b National Institute of Biological Resources, Incheon, Republic of Korea; c Department of Bioscience and Biotechnology, Konkuk University, Seoul, Republic of Korea; d Research Institute for Bioactive-Metabolome Network, Konkuk University, Seoul, Republic of Korea

**Keywords:** Reactive oxygen species, antioxidant, anti-inflammatory, lipopolysaccharide, arachidonic acid, ear oedema

## Abstract

**Context:**

*Pinus densiflora* Siebold & Zucc. (Pinaceae) needle extracts ameliorate oxidative stress, but research into their anti-inflammatory effects is limited.

**Objective:**

To investigate antioxidant and anti-inflammatory effects of a *Pinus densiflora* needles (PINE) ethanol extract *in vitro* and *in vivo*.

**Materials and methods:**

We measured levels of reactive oxygen species (ROS), superoxide dismutase (SOD) and inflammatory mediators in lipopolysaccharide (LPS)-stimulated RAW264.7 cells at various PINE concentrations (25, 50 and 100 μg/mL; but 6.25, 12.5 and 25 μg/mL for interleukin-1β and prostaglandin E_2_ (PGE_2_)). Thirty ICR mice were randomized to six groups: vehicle, control, PINE pre-treatment (0.1, 0.3 and 1 mg/left ear for 10 min followed by arachidonic acid treatment for 30 min) and dexamethasone. The posttreatment ear thickness and myeloperoxidase (MPO) activity were measured.

**Results:**

PINE 100 μg/mL significantly decreased ROS (IC_50_, 70.93 μg/mL, *p* < 0.01), SOD (IC_50_, 30.99 μg/mL, *p* < 0.05), malondialdehyde (*p* < 0.01), nitric oxide (NO) (IC_50_, 27.44 μg/mL, *p* < 0.01) and tumour necrosis factor-alpha (*p* < 0.05) levels. Interleukin-1β (*p* < 0.05) and PGE_2_ (*p* < 0.01) release decreased significantly with 25 μg/mL PINE. PINE 1 mg/ear inhibited LPS-stimulated expression of cyclooxygenase-2 and inducible NO synthase in RAW264.7 macrophages and significantly inhibited ear oedema (36.73–15.04% compared to the control, *p* < 0.01) and MPO activity (167.94–105.59%, *p* < 0.05).

**Discussion and conclusions:**

PINE exerts antioxidant and anti-inflammatory effects by inhibiting the production of inflammatory mediators. Identified flavonoids such as taxifolin and quercetin glucoside can be attributed to effect of PINE.

## Introduction

Inflammation is a complex biological response of the body to harmful stimuli, such as pathogens, reactive oxygen species (ROS), ultraviolet (UV) rays and certain cellular stimulants and defence responses, involving immune cells, blood vessels and molecular mediators. Inflammation has a beneficial function because it eliminates early causes of cytotoxicity and damaged necrotic cells and tissues that accumulate following injury and it initiates tissue repair and restoration of tissue homeostasis (Rock and Kono 2008). Acute inflammation is the body’s initial response to noxious stimuli, and is achieved by the increased movement of blood plasma and white blood cells, especially granulocytes, to the damaged tissue site. Chronic inflammation, which is a state of constant inflammation, is characterized by a gradual increase in the number of specific cell types, such as mononuclear cells, at the inflammatory sites. Simultaneous destruction and recovery of the inflamed tissue accompany the chronic inflammatory processes. Many disorders, such as atherosclerosis, myopathy, allergies and cancers, are associated with inflammation. Therefore, the inhibition of excessive inflammatory responses is the primary strategy for the treatment of inflammation-related diseases (Venkatesan et al. 2017).

Macrophages, or phagocytic cells, are one of the cell types that are responsible for immunity and are distributed in all animal tissues, wherein they engulf and eliminate aged cells and foreign organisms, such as bacteria and viruses. In addition, macrophages mediate the inflammatory response (Italiani and Boraschi 2015) and are often regarded as acute inflammatory cells because they primarily respond to inflammatory stimuli, such as interferon-gamma (IFN-γ), bacterial lipopolysaccharide (LPS), proinflammatory cytokines (tumour necrosis factor-alpha (TNF-α) and interleukin-1 beta (IL-1β)) and various other chemical mediators (Fujiwara and Kobayashi 2005). During inflammation, macrophages are activated by these cytokines and stimulants, and subsequently release various proinflammatory substances, such as TNF-α, IL-1β, IL-6, nitric oxide (NO), ROS and prostaglandin E_2_ (PGE_2_) (Akundi et al. 2005). Substances, such as ROS and cytokines, produced by excessive inflammatory reactions can damage cell membranes and tissues (Manke et al. 2013). Moreover, ROS and reactive nitrogen species (RNS) damage the lipids in cell membranes, leading to increased lipid peroxidation (LPO) (Hamza and El-Shenawy 2017). Myeloperoxidase (MPO) produced by activated polymorphonuclear neutrophils (PMNs) is a major cause of excessive oxidative stress in tissues (Tóth et al. 2017). Inflammation and oxidative stress are closely related pathophysiological events (Biswas 2016). During oxidative stress, ROS may participate in signalling of toll-like receptors (TLRs), which activate inflammatory signalling (Gill et al. 2010). Activated phagocytic cells, such as neutrophils and macrophages, produce large amounts of ROS in the inflammatory state (Fialkow et al. 2007). Due to the close relationship between oxidative stress and inflammation, the effect of therapeutic agents on oxidative stress and inflammation should be studied simultaneously.

*Pinus densiflora* Siebold & Zucc. (Pinaceae) (commonly known as the pine tree in South Korea) is widely distributed throughout Northeast Asia and accounts for 87% of Korean mountain plants. In East Asia, various parts of pine trees have been used in folk medicine and as dietary supplements (Kim and Chung 2000). Recent studies have shown the antioxidant effect of *P. densiflora* based on a superior reduction of the 1,1-diphenyl-2-picrylhydrazyl free radical compared to α-tocopherol (Jiang et al. 2012) that is attributable to high concentrations of pro-anthocyanidins, such as procyanidin, B1, B3, B7 and catechins (Park et al. 2011). Furthermore, *P. densiflora* needle extract (PINE) contains subgroups of flavonoids, such as flavonols (quercetin, kaempferol) and flavanonols (taxifolin), which exhibit considerable antibacterial and antioxidant effects (Xie et al. 2015). However, antioxidant therapy alone is unlikely to prevent diseases induced by oxidative stress, such as cardiovascular and diabetes-related complications, neurodegenerative diseases, cancer or ageing, due to the complexity of the relationship between inflammation and oxidative stress (Biswas 2016). Therefore, it is necessary to simultaneously study the effects of PINE on oxidative stress and inflammation to elucidate the effects of PINE on oxidative stress- and inflammation-related diseases. Furthermore, to date, few studies have investigated the anti-inflammatory effects of *P. densiflora*.

Therefore, this study examines the antioxidant and anti-inflammatory effects of PINE *in vitro* and *in vivo* using LPS-stimulated RAW264.7 cells and quantifies the levels of ROS, superoxide dismutase (SOD), malondialdehyde (MDA) and inflammatory cytokines (TNF-α, IL-1β, PGE_2_, cyclooxygenase 2 (COX-2) and NO) by enzyme-linked immunosorbent assay (ELISA) and Western blotting. Furthermore, an arachidonic acid-induced mouse model was used to investigate ear oedema and MPO activity *in vivo*.

## Materials and methods

### Preparation of PINE

*P. densiflora* needles were collected from Yeongdong-gun, Chungcheongbuk-do (latitude: 36°13′N, longitude: 127°55′E), Republic of Korea, in August 2014. They were identified and authenticated by National Institute of Biological Resources under Ministry of Environment. A voucher specimen of the authenticated plant material was preserved in the Wildlife Natural Products Bank of The Biological and Genetic Resources Utilization Division of the National Institute of Biological Resources (no. NIBRVP0000519857). The needles (99.5 g) were subjected to extraction three times with 70% ethanol at 20–30 °C. The resulting extract was filtered and concentrated using a rotary evaporator at 45 °C and further lyophilized to yielding 10.70 g (10.75%) of PINE. Stock solutions of the extract were prepared in dimethyl sulphoxide (DMSO; D8418, Sigma-Aldrich, Seoul, Korea) at concentrations of 20 and 100 mg/mL for cell and animal experiments, respectively.

### Ultrahigh-performance liquid chromatography-Q Extractive-Orbitrap-mass spectrometry (UHPLC-Q-Orbitrap-MS) analysis of PINE

The metabolite analysis of PINE was conducted via UHPLC-Q-Orbitrap-MS, as described previously (Lee et al. 2020). For the analysis, lyophilized PINE was re-dissolved in 70% ethanol and filtered through a 0.2 μm polytetrafluoroethylene filter (Chromdisc, Daegu, Korea). Samples were separated on a Hypersil Gold C18 Selectivity LC Column (i.d., 1.9 μm, 50 × 2.1 mm; Thermo Fisher Scientific, Waltham, MA) at a column oven temperature of 25 °C. The mobile phases comprised 0.1% formic acid in water (B) and in acetonitrile (C), and the compositions of the gradient flows were the same. The gradient was gradually increased from 0% solvent C to 100% solvent (C) over 20 min and was maintained for another 2 min at a flow rate of 0.3 mL/min with a 10 μL injection volume. Mass spectra were obtained using electrospray ionization in negative and full-scan modes within 100–1000 *m/z* under the following operating conditions: spray needle voltage, ±3.3 kV; capillary temperature, 320 °C; probe heater temperature, 300 °C; stacked ring ion guide (S-lens) radiofrequency (RF) level, 60%; resolution (full-width at half-maximum; FWHM), 35,000.

### Cell culture

The murine macrophage RAW264.7 cell line was purchased from the American Type Culture Collection (ATCC, Manassas, VA), cultured in Dulbecco's modified Eagle medium (WELGENE Inc., Gyeongsan-si, South Korea), and supplemented with 10% foetal bovine serum (WELGENE Inc., Gyeongsan-si, South Korea) and a 1% antibiotic–antimycotic solution (Gibco, Waltham, MA) in a 5% CO_2_ incubator at 37 °C. For treatment, the stock solution of PINE was initially added to the culture medium in the test tube, mixed thoroughly, and the DMSO concentration was adjusted to 0.5% in all culture mediums. Then, the cell culture medium was replaced with the mixed medium.

### Measurement of cell viability

To evaluate the cell toxicity of PINE, we performed the 3-[4,5-dimethylthiazol-2-yl]-2,5-diphenyltetrazolium bromide (MTT) colorimetric assay (Woerdenbag et al. 1994). RAW264.7 macrophages were seeded in 96-well plates (2 × 10^5^ cells/well) and incubated at 37 °C in a 5% CO_2_ incubator for 24 h. After removing the medium, fresh mediums with PINE at various concentrations (25, 50 or 100 μg/mL) were added to the wells, and the cells were incubated for another 24 h. Subsequently, the medium in each well was replaced with 200 μL 0.5 mg/mL MTT dissolved in phosphate-buffered saline (PBS). The cells were incubated for 4 h, and the supernatant was carefully replaced with 200 μL DMSO to dissolve the MTT formazan, and absorbance was measured at 570 nm using a microplate reader (the number of replicates; *n* = 3–4) (Flexstation 3, Molecular Devices, San Jose, CA).

### ROS scavenging activity

RAW264.7 cells were cultured for 24 h in 96-well plates and pre-treated with PINE (25, 50 or 100 μg/mL) for 3 h, followed by treatment with 1 μg/mL LPS in a fresh medium and 24-incubation. Next, the cells were treated with 20 μM 2′,7′-dichlorodihydrofluorescein diacetate (H_2_DCF-DA) for 30 min at 37 °C. ROS were detected using a fluorescence microplate reader (Flexstation 3, Molecular Devices, San Jose, CA) at excitation and emission wavelengths of 485 and 535 nm, respectively. Representative fluorescence images were captured using a Leica DM 480 camera (Leica, Wetzlar, Germany) (*n*= 9).

### LPS-induced SOD production

SOD levels in the lysates of the cultured cells were quantified using an ELISA kit in accordance with the manufacturer's protocol (Cloud‐Clone Corp., Katy, TX). Briefly, RAW264.7 cells were cultured for 24 h in 24-well plates and pre-treated with PINE (25, 50 or 100 μg/mL) for 3 h, then stimulated with 1 μg/mL LPS for 24 h, and, after washing with PBS, incubated with 400 μL radioimmunoprecipitation assay (RIPA) buffer (Thermo Fisher Scientific, Waltham, MA) per well for 5 min. The lysates were centrifuged at 12,000×*g* for 15 min, and the supernatants were collected to measure the SOD levels (*n*= 3).

### Thiobarbituric acid-reactive substances (TBARS) assay

RAW264.7 macrophages were cultured in a 100-mm dish (2 × 10^6^ cells), washed with PBS, harvested using a scraper, transferred to Eppendorf (EP) tubes at 2 × 10^5^ cells/tube, and treated with PINE (25, 50 or 100 μg/mL) for 30 min at 37 °C. The cells were centrifuged at 750×*g* for 10 min, and the supernatants were removed. The pellets were resuspended in 1 μM hydrogen peroxide (H_2_O_2_) and 10 mM ferrous sulphate (FeSO_4_) in PBS for 15 min and then sonicated (*n*= 3). In the animal model, the ear tissues were homogenized in the homogenizing solution (80 mM sodium phosphate, 0.5% hexadecyltrimethylammonium bromide, pH 5.4) and sonicated. The homogenates were centrifuged at 11,200×*g* for 20 min at 4 °C, and the supernatant was transferred to EP tubes (*n*= 4–5). The absorbance of thiobarbituric acid was used to measure the extent of LPO (Boland et al. 2000). Trichloroacetic acid was added to the EP tubes at a final concentration of 5%, an equal volume of 0.325% (w/v) 2-thiobarbituric acid in 50% acetic acid was added, and the mixture was incubated at 95 °C for 30 min. After cooling in an ice bath, 500 μL 2-methylpropan-1-ol was added to each EP tube, and the tubes were vigorously mixed. The tubes were then centrifuged at 750×*g* for 10 min, and 200 μL of the upper organic phase was transferred from each tube to a well of a 96-well plate. The absorbance of each well was measured using a microplate reader at 532 nm and converted to the corresponding MDA concentrations using a standard curve of serially diluted MDA. In this assay, caffeic acid was used as a reference substance because of its ability to inhibit LPO when evaluated by TBARS analysis (Pari and KarthiKesan 2007; Oboh et al. 2021).

### Measurement of nitric oxide levels

RAW264.7 macrophages were seeded in 96-well plates (2 × 10^5^ cells/well) and incubated at 37 °C in a 5% CO_2_ incubator for 24 h. Each well was pre-treated with the test agent for 1 h. The cells were stimulated with 1 μg/mL LPS for 24 h. Subsequently, 100 μL each of both supernatant and modified Griess reagent (Sigma-Aldrich, St. Louis, MO) were added to each well, and the plates were incubated at 20–30 °C in the dark for 10 min. The absorbance of each well was measured at 550 nm using a microplate reader. Nitrite concentrations were calculated using a standard curve of serially diluted sodium nitrite (*n*= 3–4).

### Western blotting analysis

RAW264.7 macrophages were seeded in six-well plates (2 × 10^5^ cells/well) and incubated at 37 °C in a 5% CO_2_ incubator for 24 h. Each well was pre-treated with the test agent for 1 h. Cells were cultured with 1 μg/mL LPS for 12 h. To prepare whole-cell lysates, each well was washed with PBS and then scraped and centrifuged at 17,000×*g* for 20 min at 4 °C. After the supernatant was removed, the pellet was resuspended in 100 μL RIPA buffer (Thermo Fisher Scientific, Waltham, MA) containing a protease inhibitor cocktail (cOmplete Tablets EDTA-free EASYpack; Roche, Basel, Switzerland) and incubated in an ice bath for 1 h. Cell lysates were centrifuged at 17,000×*g* for 20 min at 4 °C, and the supernatants containing cytosolic proteins were collected for Western blotting. The total protein concentration was measured using the BCA protein assay reagent (Thermo Fisher Scientific, Waltham, MA). Equal amounts of protein samples were resolved by 10% sodium dodecyl sulphate-polyacrylamide gel electrophoresis (SDS-PAGE) and electro-transferred to polyvinylidene fluoride (PVDF; Merck, Branchburg, NJ) membranes, which were subsequently incubated with 5% bovine serum albumin in TBS-T buffer (50 mM Tris–HCl, 150 mM NaCl and 0.1% Tween 20, pH 7.6) for 2 h at room temperature to block non-specific protein binding, and further incubated overnight at 4 °C with the following primary antibodies: anti-COX-2 (R&D Systems, Minneapolis, MN), anti-inducible nitric oxide synthase (iNOS; Upstate Biotechnology, Lake Placid, NY) and anti-β-actin (Santa Cruz Biotechnology, Dallas, TX). Thereafter, the membranes were incubated with the appropriate horseradish peroxidase (HRP)-conjugated secondary antibodies (Thermo Fisher Scientific, Waltham, MA) for 2 h at room temperature. Enhanced chemiluminescence substrate (Western Lightning ECL Pro; PerkinElmer Inc., Waltham, MA) was used to develop protein bands, the signal was detected using Chemidoc XRS (Bio-Rad, Hercules, CA), and the signal intensity was analysed using Quantity One software (Bio-Rad, Hercules, CA) and normalized with β-actin as a housekeeping protein. Each quantification data point is expressed relative to the control. Western blotting was performed in triplicate (*n*= 3).

### Measurement of TNF-α, IL-1β and PGE2 levels via ELISA

RAW264.7 macrophages were seeded in six-well plates (2 × 10^5^ cells/well) and incubated at 37 °C in a 5% CO_2_ incubator for 24 h. Each well was pre-incubated with the test agent for 1 h. The cells were then stimulated with 1 μg/mL LPS for 12 h. The supernatants were transferred to EP tubes and centrifuged at 17,000×*g* for 10 min at 4 °C and used to determine the levels of TNF-α, IL-1β and PGE_2_ by ELISA. Each cytokine was quantified according to the protocol of each kit (R&D Systems, Minneapolis, MN) (*n*= 3).

### Experimental animals

Institute of Cancer Research (ICR) mice (male, 6 weeks old) were purchased from Young Bio (Young Bio Inc., Seongnam, Republic of Korea) and housed in the animal facility of Chung-Ang University in a standard, environmentally controlled animal room (temperature: 24 ± 2 °C, humidity: 50 ± 5%, illumination: 300–500 lux) with free access to pathogen-free food and water *ad libitum*. After acclimatization for 1 week, the mice were randomly assigned to six groups containing five mice each (control, arachidonic acid-treated group, arachidonic acid + PINE 0.1 mg/ear, arachidonic acid + PINE 0.3 mg/ear, arachidonic acid + PINE 1 mg/ear and arachidonic acid + dexamethasone). All experimental procedures complied with the guidelines established by the Institutional Animal Care and Use Committee of Chung-Ang University, and the study design was approved by the appropriate ethics review board (IACUC 2020-00135).

### Arachidonic acid-induced ear oedema model

To induce acute inflammation, arachidonic acid was selected as a stimulant based on previous research (Young et al. 1984; Yu et al. 2020) and applied to the left ear of the mice. Before applying arachidonic acid, the test agent (100 mg/mL in DMSO stock solution) was prepared at 2, 6 and 20 mg/mL concentrations (diluted in 10% ethanol). Dexamethasone (100 mg/mL in DMSO stock solution) was prepared at a 2 mg/mL concentration using 10% ethanol. The ratio of all vehicles (DMSO: 10% ethanol) was set as 1:4. In the drug-treatment groups, 50 μL test agent (0.1, 0.3 and 1 mg/ear) and dexamethasone (0.1 mg/ear) were applied to the inside of the left ear (∼1 cm^2^ area) and left for 10 min. In the control and arachidonic acid-treated groups, a vehicle was applied to the inside of the left ear instead of the test agents. Moreover, the vehicle was applied to the right ear of all groups for comparison of the left and right ears to verify that the vehicle combined with DMSO and 10% ethanol is not interfering the effect of PINE. Twenty microliter of the arachidonic acid solution (100 mg/mL in acetone) was applied to each left ear for 30 min to induce inflammation while the right ear received 20 μL of acetone to verify that this solvent is not interfering the ear inflammation. In the case of the control group, 20 μL of acetone was applied to both ears to verify that this solvent is not interfering the ear inflammation. Thereafter, mice were anaesthetized by intramuscular injection with 20 mg/kg of Zoletil 50 (125 mg tiletamine and 125 mg zolazepam; Virbac, Carros, France) and 5 mg/kg xylazine (Sigma-Aldrich, St. Louis, MO), and the thickness of the ear was measured using a micro-engineer metre (Mitutoyo, Kawasaki-shi, Japan). The mice were euthanized by CO_2_ asphyxiation, and the ear tissues were stored at −80 °C until analysis.

### MPO activity assay

MPO activity in the ear tissues was assessed using colorimetry. The ear tissues were homogenized in a homogenizing solution (80 mM sodium phosphate, 0.5% hexadecyltrimethylammonium bromide, pH 5.4) and sonicated, the homogenates were centrifuged at 11,200×*g* for 20 min at 4 °C, and 30 μL of the supernatant was transferred to each well of a 96-well plate. Next, 200 μL of the reaction mixture (150 mM sodium phosphate; 0.01% H_2_O_2_, pH 5.4) was added to each well, and 20 μL 18.4 mM tetramethylbenzidine in dimethylformamide was added to each well to initiate the reaction. The samples were then incubated for 3 min at 37 °C (Vasconcelos et al. 2017). After the samples were cooled on ice, 30 μL 1.46 M sodium acetate buffer (pH 3.0) was added to each well to stop the reaction, and absorbance was measured at 620 nm using a microplate reader (*n*= 3).

### Histological analysis of ear tissues

The ear tissues were removed from each mouse, fixed with 10% formalin solution, embedded using Tissue-Tek^®^ (Sakura Finetek^®^, Torrance, CA), and then sectioned using a Leica microtome 820 (Leica Microsystems, Wetzlar, Germany) at 4 µm (*n* = 3). The sectioned tissues were stained with haematoxylin–eosin (H&E). Six random fields of each stained tissue section were observed under a microscope (Leica Microsystems, Wetzlar, Germany), and images were captured using a Leica DM 480 camera (Leica Microsystems, Wetzlar, Germany) and processed using Leica Application Suite (LAS) software (Leica Microsystems, Wetzlar, Germany).

### Statistical analysis

Statistical analyses were conducted using one-way analysis of variance (ANOVA) followed by Tukey’s *post hoc* test. For homogeneity testing, the Brown–Forsythe and Bartlett’s tests were performed. The Shapiro–Wilk test was performed to test for normality. Differences were considered significant at *p* < 0.05.

## Results

### Phytochemical analysis of PINE

**Figure 1.**
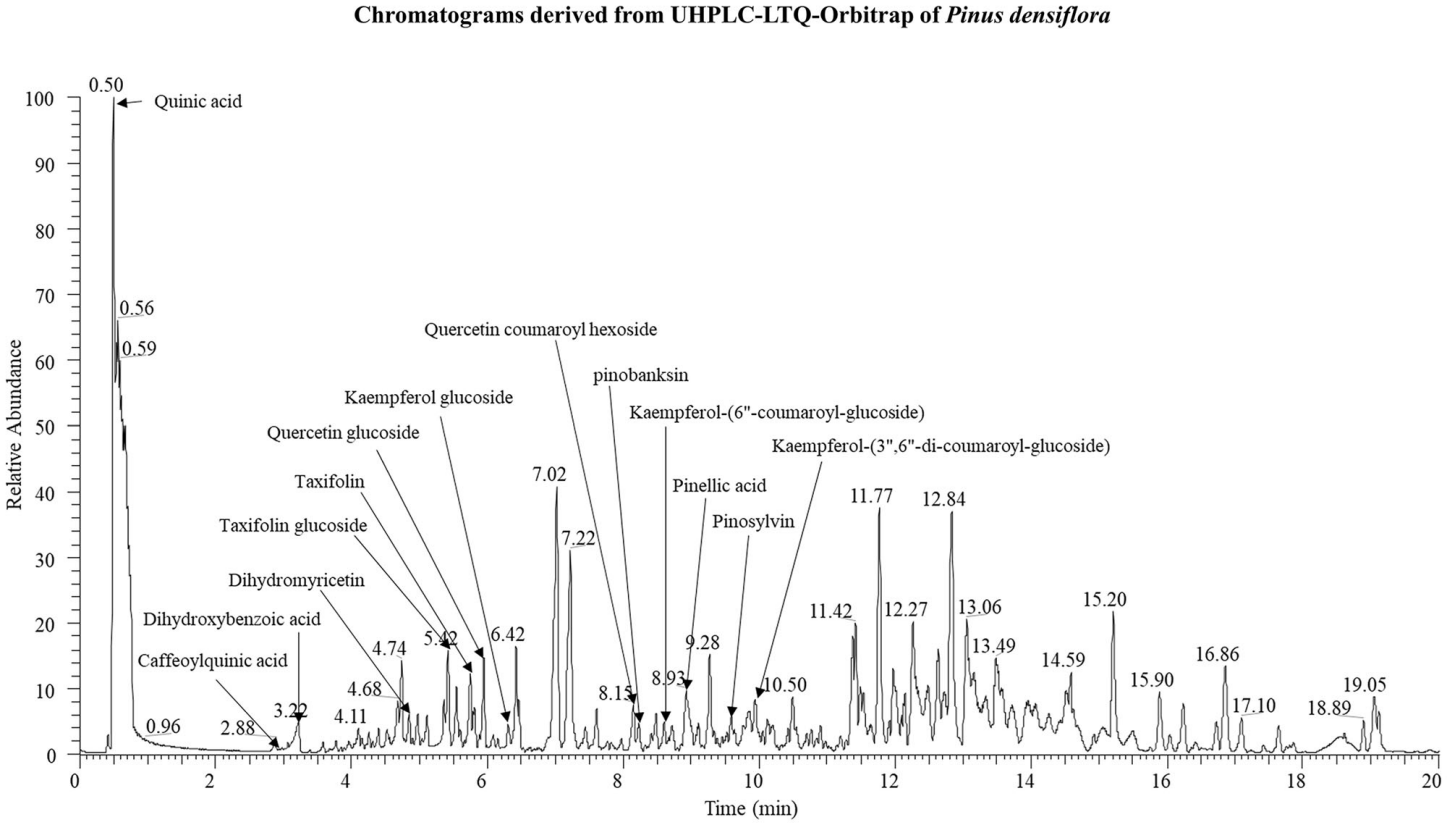
Chromatogram derived from UHPLC-LTQ-Orbitrap analysis of *P. densiflora* extract.

Phytochemical analysis of PINE putatively identified a total of 14 metabolites, including two organooxygen (quinic acid and caffeoylquinic acid), one benzene (dihydrobenzoic acid), nine flavonoids (pinobanksin, kaempferol glucoside, kaempferol 6″-coumaroyl-glucose, kaempferol 3″,6″-dicoumaroyl-glucoside, quercetin glucoside, quercetin coumaroyl hexoside, taxifolin, taxifolin glucoside and dihydromyricetin), one stilbene (pinosylvin) and one long-chain fatty acid (pinellic acid). These tentatively identified metabolites were annotated based on their retention times, and tandem mass spectrometric fragment patterns, with reference to data from published studies. These metabolites are marked on the chromatograms derived from the UHPLC-LTQ-Orbitrap-MS analysis (Figure 1 and Table 1).

**Table 1.** ID table of *Pinus densiflora* needle extract.

Tentative identifications	Ret^a^	Measured mass (*m/z*)^b^		M.W.^c^	Elemental composition [M‒H]^‒^	Δ ppm^d^	[M‒H]^‒^ MS*^n^* fragments (*m/z*)	I.D.^e^
		[M‒H]^‒^	[M + H]^+^					
Quinic acid	0.50	191.0560	193.0633	192.1666	C_7_H_11_O_6_	–0.426	191 > 173, 111, 93, 87 > 85, 83	STD^f^ and REF^g^ (Karapandzova et al. 2015)
Caffeoylquinic acid	2.95	353.0877	355.1017	354.3087	C_16_H_17_O_9_	–0.185	353 > 191, 173, 135 > 155, 111	REF^g^ (He et al. 2010)
Dihydroxybenzoic acid	3.22	153.0182	155.0344	154.1210	C_7_H_5_O_4_	–0.045	153 > 122, 108, 107 > 80, 66	REF^g^ (Shadkami et al. 2007)
Pinobanksin	8.27	271.0613	273.0757	272.2528	C_15_H_11_O_5_	0.418	271 > 253, 225, 127 > 221, 197	REF^g^ (Pietta et al. 2002; Castro et al. 2014)
Kaempferol glucoside	6.30	447.0935	449.1079	448.3780	C_21_H_19_O_11_	0.348	447 > 327, 284 > 255, 227	STD and REF^g^ (Slimestad 2003)
Kaempferol-(6″-coumaroyl-glucoside)	8.60	593.1309	595.1450	594.5196	C_30_H_25_O_13_	1.342	593 > 447, 301, 285 > 301	REF^g^ (Slimestad 2003)
Kaempferol-(3″,6″-di-coumaroyl-glucoside)	9.94	739.1682	741.1814	740.6624	C_39_H_31_O_15_	1.687	739 > 593, 301, 285 > 447, 301	REF^g^ (Slimestad 2003)
Quercetin glucoside	5.89	463.0888	465.1031	464.3763	C_21_H_19_O_12_	1.103	463 > 301 > 273, 229, 179	STD and REF^g^ (Slimestad 2003)
Quercetin coumaroyl hexoside	8.17	609.1256	611.1393	610.5190	C_30_H_25_O_14_	0.938	609 > 445, 301, 255 > 178	REF^g^ (Abou-zaid and Nozzolillo 1991; Candela et al. 2020)
Taxifolin	5.80	303.0510	305.0656	304.2540	C_15_H_11_O_7_	0.013	303 > 285, 217, 179, 151, 125	REF^g^ (Slimestad 2003; Dias et al. 2013; Gabaston et al. 2017)
Taxifolin glucoside	5.42	465.1039	467.1186	466.3950	C_21_H_21_O_12_	0.066	465 > 437, 361, 303, 285	REF^g^ (Slimestad 2003)
Dihydromyricetin	4.85	319.0459	321.0606	320.2516	C_15_H_11_O_8_	–0.221	319 > 301, 257, 215, 193, 151, 125	REF^g^ (Slimestad 2003; Fan et al. 2017; Park et al. 2017)
Pinosylvin	9.62	211.0757	213.0910	212.2439	C_14_H_11_O_2_	–3.378	211 > 167, 153, 137, 108	STD and REF^g^ (Yeo et al. 2013; Gabaston et al. 2017)
Pinellic acid	8.93	329.2335	353.2300^h^	330.4596	C_18_H_33_O_5_	0.160	329 > 293, 229, 183, 171 > 211, 155, 125	REF^g^ (Barragán-Zarate et al. 2020)

^a^ Retention time.

^b^ Accurate mass spectra (mass-to-charge, *m/z*) from electrospray ionization (ESI) positive and negative modes analysed by UHPLC-Q-Orbitrap-MS.

^c^ Molecular weight.

^d^ Mass tolerance from elemental composition analysis.

^e^ Identifications.

^f^ Standard.

^g^ Reference.

^h^ [M + Na]^+^.

### PINE cytotoxicity in RAW264.7 cells

**Figure 2.**
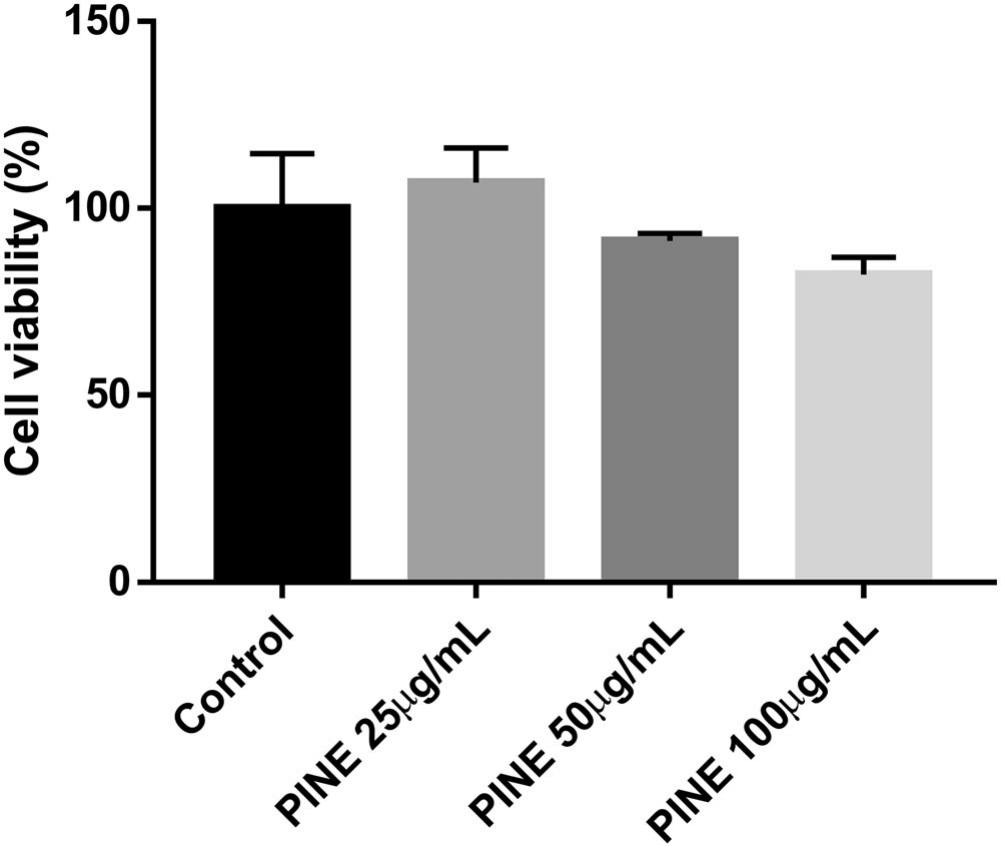
Cytotoxicity of PINE towards RAW264.7 macrophages. RAW264.7 macrophages were incubated with various concentrations of PINE for 24 h at 37 °C. The number of viable cells was assessed via MTT assay. The viability of the control group cells was considered to be 100%. Each value represents the mean ± SD of four separate experiments.

The MTT assay was performed to determine the toxicity of PINE for RAW264.7 cells. At PINE concentrations of 25, 50 and 100 µg/mL, the viability of the RAW264.7 cells was 110.189 ± 1.205%, 110.379 ± 1.294% and 86.151 ± 2.721%, respectively, with regard to the untreated control (Figure 2). Thus, PINE showed no significant cytotoxicity at 25, 50 or 100 µg/mL, but demonstrated cytotoxicity at concentrations greater than 100 µg/mL. Therefore, we selected 25, 50 and 100 µg/mL PINE doses for subsequent experiments (Figure 2).

### Effects of PINE on oxidative stress and NO generation in RAW264.7 cells

**Figure 3.**
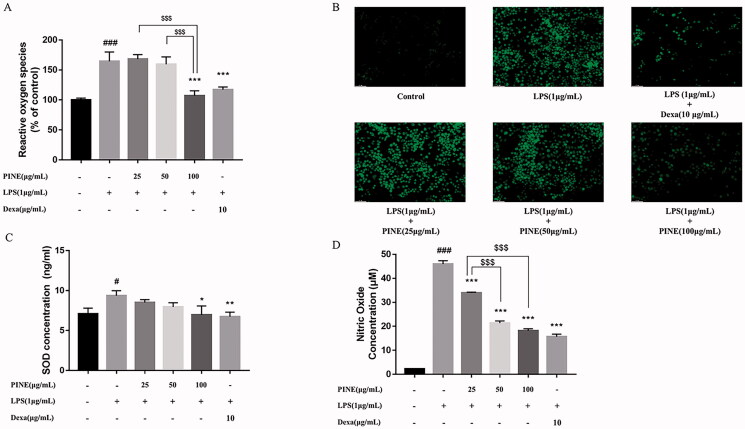
Effects of PINE on oxidative stress and NO production in RAW264.7 cells. (A, B) ROS levels were measured via DCF-DA assay, and fluorescence was detected using a microplate reader (excitation wavelength, 485 nm; emission wavelength, 535 nm). (C) SOD concentration was measured via ELISA. (D) NO level was measured using the Griess reagent. Stimulation was performed by LPS (1 μg/mL) treatment, and the cells were pre-treated with various concentrations of PINE (25, 50 or 100 μg/mL). Dexamethasone (Dexa; 10 μg/mL) was used as the reference compound. Results are expressed as activity percentages, and each value represents the mean ± SD of four separate experiments. Significantly different from the control (^#^*p* < 0.05 and ^###^*p* < 0.001); significantly different from the stimulation group (*p < 0.05, **p < 0.01 and ***p < 0.001); and significant intergroup differences (^$$$^*p* < 0.001).

To investigate the antioxidant effects of PINE on RAW264.7 cells, we measured ROS and SOD levels (Figure 3(A–C)). LPS treatment significantly elevated ROS and SOD levels, and 100 µg/mL PINE significantly suppressed ROS generation (IC_50_: 70.93 μg/mL). At 50 and 100 µg/mL, PINE significantly reduced SOD levels (IC_50_: 30.99 μg/mL). Dexamethasone (10 μg/mL) was used as a reference compound because of its ability to significantly inhibit LPS-induced elevations in ROS and SOD concentrations.

To confirm the effects of PINE on LPS-induced inflammatory responses in RAW264.7 cells, we measured levels of NO, which is a proinflammatory mediator (Figure 3(D)), and found that PINE inhibited NO production in a concentration-dependent manner. NO levels in LPS-treated cells were approximately nine times that in the negative control cells. Pre-treatment with 25, 50 and 100 μg/mL PINE suppressed NO production to 26.18%, 53.49% and 60.42%, respectively, in LPS-treated cells (IC_50_: 27.44 μg/mL). Dexamethasone (10 μg/mL), the reference compound, inhibited NO production to 65.82% in the LPS-treated cells.

### Effects of PINE on TBARS in RAW264.7 macrophages

**Figure 4.**
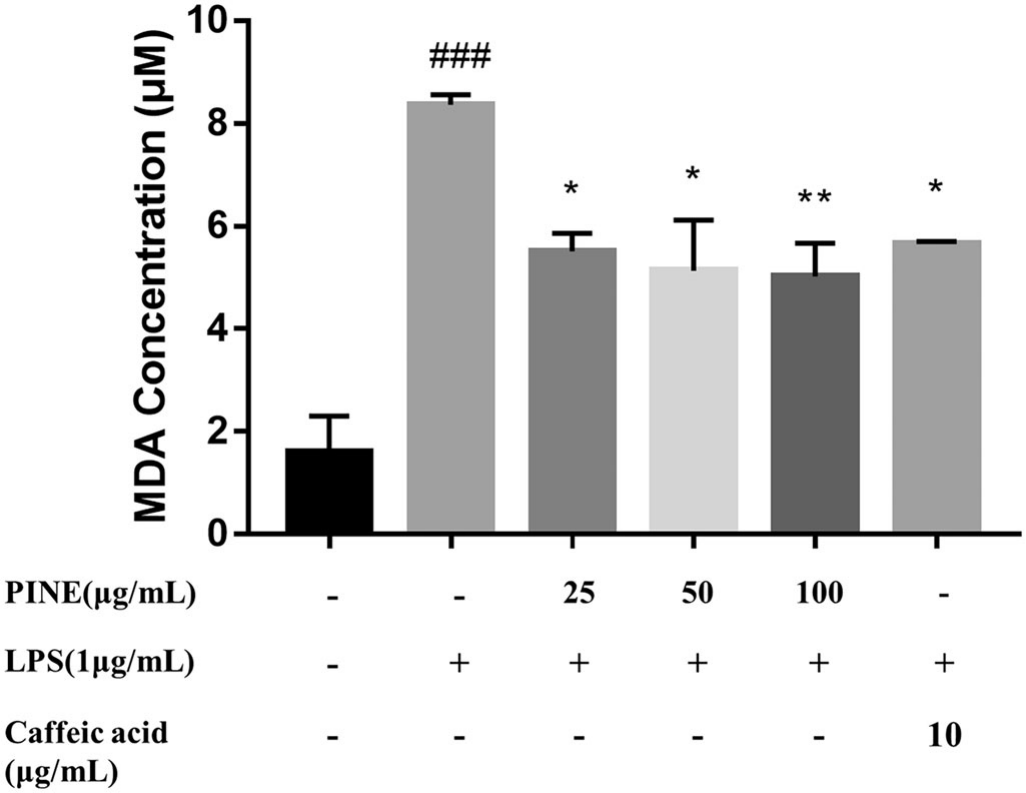
Effects of PINE on lipid peroxidation in RAW264.7 macrophages. Cells were pre-treated with PINE (25, 50 or 100 μg/mL) for 30 min and then incubated with 1 μM H_2_O_2_ and 10 mM FeSO_4_ for 15 min to induce lipid membrane peroxidation, followed by sonication. Thiobarbituric acid reacts with MDA to generate a red adduct, which can be quantitated by measuring the absorbance at 532 nm. Caffeic acid (25 μg/mL) was used as the reference compound. Results are expressed as an MDA concentration, and each value represents the mean ± SD of four separate experiments. Significantly different from the control (^###^*p* < 0.001); significantly different from the stimulation group (*p < 0.05 and **p < 0.01).

To investigate the effects of PINE on LPO in RAW264.7 cells, we measured levels of MDA, an end product of the degradation of LPO products. Treatment with 25, 50 and 100 µg/mL PINE significantly suppressed LPO to 34.12%, 38.7% and 40.0%, respectively, in the oxidative reagent-treated group. The antioxidant used as the reference compound, caffeic acid (25 μg/mL), exerted significant inhibition by decreasing the LPO levels to 32.44% in the oxidative reagent-treated group (Figure 4).

### Effects of PINE on LPS-induced iNOS and COX-2 protein levels in RAW264.7 macrophages

**Figure 5.**
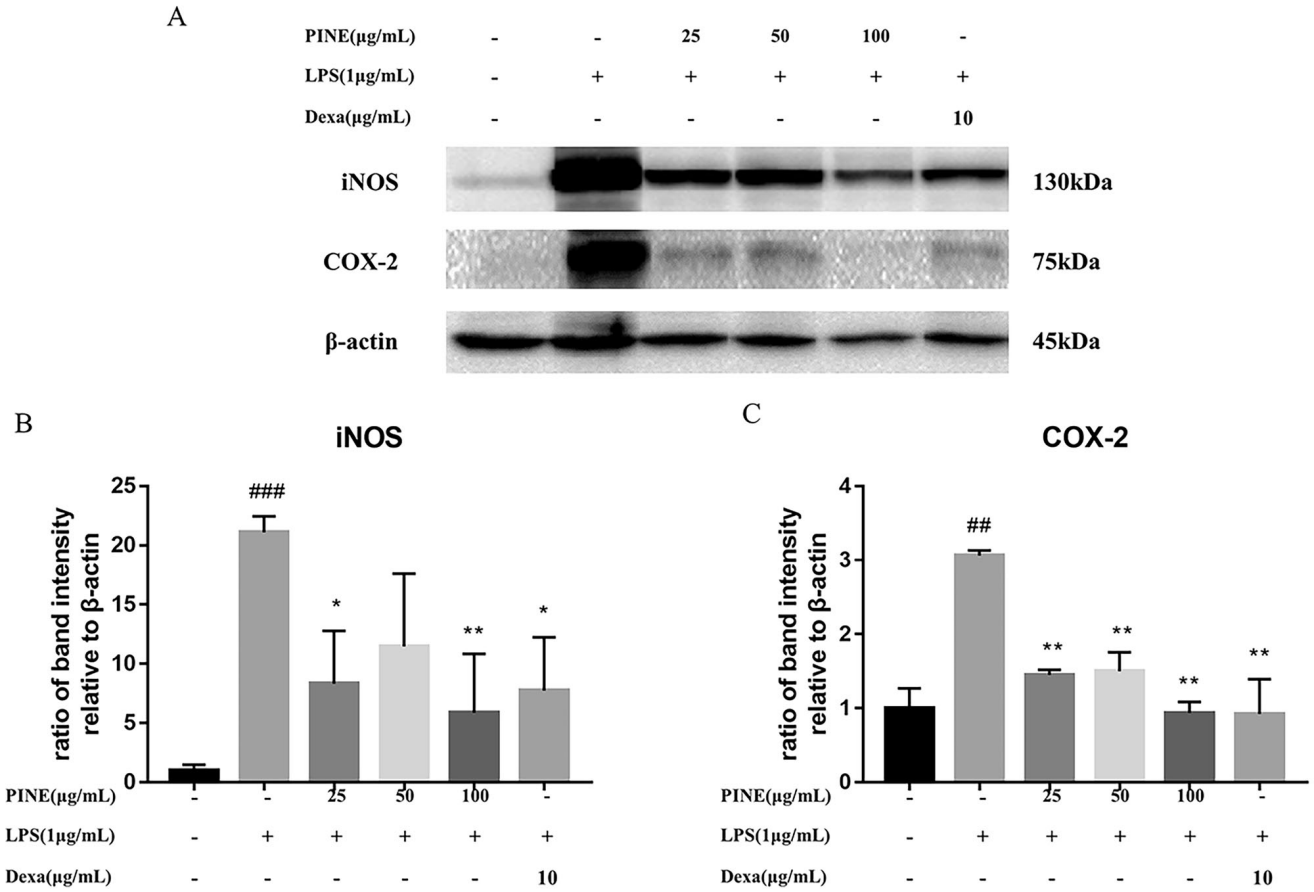
Effects of PINE on LPS-induced iNOS and COX-2 protein levels in LPS-stimulated RAW264.7 macrophages. Western blotting analysis for iNOS, COX-2 and β-actin levels. Cells were pre-treated with PINE (25, 50 or 100 μg/mL) for 1 h and then treated with LPS (1 μg/mL) for 12 h. Dexamethasone (Dexa; 10 μg/mL) was used as the reference compound. The results were analysed using Chemidoc XRS and Quantity One. Experiments were repeated four times. Significantly different from the control group (^##^p < 0.01 and ^###^*p* < 0.001); significantly different from the stimulation group (*p < 0.05 and **p < 0.01).

LPS-stimulated macrophages produce cytokines, chemokines and proteins, such as iNOS and COX-2, which produce the important inflammatory mediators NO and PGE_2_. We used Western blotting to examine the effects of PINE on the levels of the abovementioned proteins in LPS-induced RAW264.7 macrophages. PINE decreased the synthesis of iNOS and COX-2 proteins relative to that observed in the LPS-treated cells (Figure 5). Interestingly, negligible levels of iNOS and COX-2 proteins were detected in the control group. Cells treated with a high PINE concentration (100 μg/mL) or dexamethasone (10 μg/mL) showed protein levels that were similar to those of the untreated cells (Figure 5).

### Effects of PINE on TNF-α, IL-1β and PGE2 production in RAW264.7 macrophages

We estimated the effects of PINE on the release of TNF-α, IL-1β and PGE_2_, which are proinflammatory mediators. The total amount of TNF-α generated in the LPS-treated cells was approximately seven times that in the untreated cells. In PINE-treated cells, TNF-α production was significantly inhibited (to 32.88% that of LPS-treated cells) only when pre-treated with a high concentration of 100 μg/mL PINE (Table 2). Table 3 shows significant inhibition in IL-1β production by cells treated with 25 μg/mL PINE. PGE_2_ production was significantly suppressed in cells treated with 12.5 and 25 μg/mL PINE (Table 3). Dexamethasone, the reference compound, showed significant inhibitory activity towards TNF-α, IL-1β and PGE_2_ production in RAW264.7 cells.

**Table 2.** Effects of *P. densiflora* needle extract (PINE) on TNF-α level in LPS-stimulated RAW264.7 macrophages.

		LPS, 1 μg/mL				
	Control	Vehicle	PINE, 25 μg/mL	PINE, 50 μg/mL	PINE, 100 μg/mL	Dexa, 10 μg/mL
TNF-α	9.93 ± 3.05	119.25 ± 4.34^##^	110.73 ± 13.82	119.02 ± 8.52	80.05 ± 7.39*	60.95 ± 1.29**

Cells were pre-treated with PINE (25, 50 or 100 μg/mL) for 1 h and treated with LPS (1 μg/mL) for 12 h. The ELISA was performed to measure the levels of TNF-α. Dexamethasone (Dexa) (10 μg/mL) was used as the reference compound. The absorbance was measured at 450 nm, and each value represents the mean ± SD (*n* = 3). Significantly different from the control (^##^*p* < 0.01). Significantly different from the stimulation group (*p < 0.05, **p < 0.01).

**Table 3.** Effects of *P. densiflora* needle extract (PINE) on the levels of IL-1β and PGE_2_ in LPS-stimulated RAW264.7 macrophages.

		LPS 1 μg/mL + melittin 0.5 μM				
	Control	Vehicle	PINE 6.25 μg/mL	PINE 12.5 μg/mL	PINE 25 μg/mL	Dexa 10 μg/mL
IL-1β	2.97 ± 2.36	18.94 ± 1.77^#^	20.33 ± 0.59	19.50 ± 1.77	10.61 ± 0.98*	5.33 ± 1.37*
PGE2	11.79 ± 4.30	904.73 ± 60.59^#^	778.82 ± 22.37	321.50 ± 30.73**	160.16 ± 16.84**	148.38 ± 8.52**

Cells were pre-treated with PINE (6.25, 12.5 or 25 μg/mL) for 1 h and then treated with LPS (1 μg/mL) and melittin (0.5 μM) for 12 h. The ELISA was performed to measure the levels of IL-1β and PGE_2_. Dexamethasone (Dexa) (10 μg/mL) was used as the reference compound. The absorbance was measured at 450 nm, and each value represents the mean ± SD (*n* = 3). Significantly different from the control (^#^*p* < 0.05). Significantly different from the stimulation group (*p < 0.05 and **p < 0.01).

### Effects of PINE on arachidonic acid-induced ear oedema in mice

**Figure 6.**
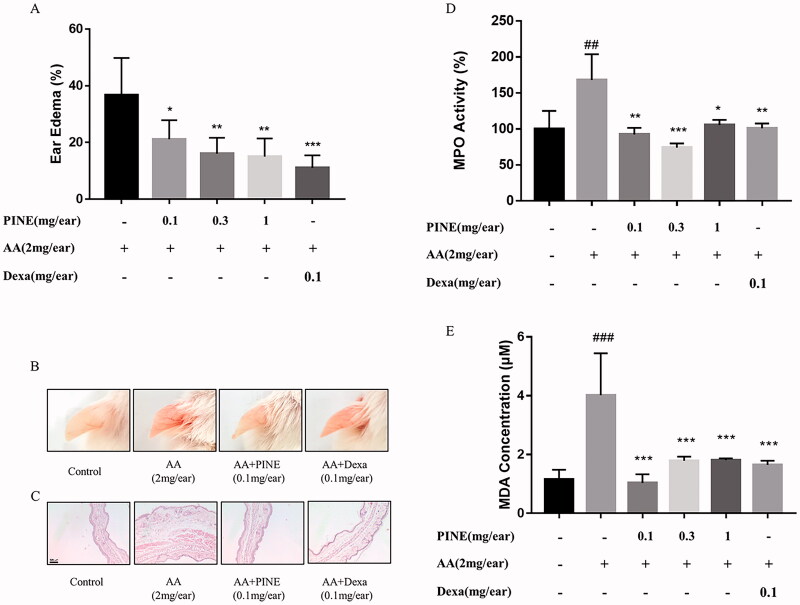
Effects of PINE on arachidonic acid-induced ear oedema in mice. (A) PINE (0.1, 0.3 or 1 mg/ear in ethanol) was applied to the inside of the left ears of ICR mice and given 10 min to absorb. Arachidonic acid (AA; 2 mg/ear dissolved in acetone) was applied for 30 min to the left ears. Dexamethasone (Dexa; 0.1 mg/ear) was applied as the reference compound to the left ear in the Dexa group. Ear thickness was measured using the micro-engineer metre. (B) Representative images of mouse ears and (C) H&E staining for each experimental group (magnification, ×10; scale bar, 100 μm). (D) MPO activity was measured in the ear tissues via colorimetry based on the absorbance at 620 nm. (E) The MDA level was quantified in the ear tissues via the TBARS method. Each value represents the mean ± SD of three separate experiments. Significantly different from the AA treatment group (*p < 0.05, **p < 0.01 and ***p < 0.001). Significantly different from the control (^##^*p* < 0.01 and ^###^*p* < 0.001).

To investigate the anti-inflammatory effects of PINE in an animal inflammation model, we measured the effects of PINE on arachidonic acid-induced ear oedema in mice. When mice were treated with arachidonic acid, the oedema rate was significantly higher than that in the control group. The PINE-treated groups at the concentrations of 0.1, 0.3 and 1 mg per ear showed significant inhibition of ear oedema. Of note, ear oedema in the PINE-treated group at the concentration of 1 mg/ear was decreased from 36.73% to 15.04%. Dexamethasone, which was used as a reference compound, significantly reduced arachidonic acid-induced ear oedema (Figure 6(A,B)).

Using H&E staining, we estimated the effects of PINE on histological changes. Arachidonic acid induced oedema of ear tissues; the PINE treatment group showed less oedema than the arachidonic acid treatment control group. Dexamethasone, in the positive control group, inhibited arachidonic acid-induced histological changes (Figure 6(C)). MPO activity was assessed as a marker of inflammation in mouse ear tissues. In the arachidonic acid-treated group, MPO activity increased 1.5-fold upon induction, but the PINE-pre-treated groups (0.1 and 0.3 mg/ear) showed significant inhibition of MPO activity. Dexamethasone, the reference compound, significantly inhibited arachidonic acid-induced MPO activation (Figure 6(D)). MDA levels, evaluated using TBARS assay, showed an approximately four-fold increase in the arachidonic acid-induced group compared to the control group. PINE treatment (0.1, 0.3 and 1 mg/ear) significantly decreased arachidonic acid-induced MDA production. Dexamethasone, as a positive control, showed significant inhibition of arachidonic acid-induced MDA production (Figure 6(E)).

## Discussion

*P. densiflora* is widely distributed throughout the Korean mountains. Recent studies have shown that *P. densiflora* has skin-protective, anti-ageing, anti-obesity, antioxidant and DNA-protective effects (Jiang et al. 2012; Jung et al. 2014; Ahn and Go 2017). Based on the results of *in vitro* and *in vivo* experiments, this study showed that PINE decreases inflammatory and oxidative responses. In this study, we also found that PINE contains large amounts of quinic acid, which has potent antioxidant properties and anti-neuroinflammatory activity (Jang et al. 2017). PINE also contains taxifolin, taxifolin glucoside and quercetin glucoside, which have antioxidant, anti-proliferative and anti‐inflammatory properties (Tóth et al. 2017; Xie et al. 2017; Lee et al. 2019).

Oxidative stress is associated with ageing and several chronic diseases, such as cardiovascular disease, chronic obstructive pulmonary disease, chronic kidney disease, neurodegenerative disease and cancer (Liguori et al. 2018). In this study, LPS-induced ROS production was significantly suppressed by PINE. SODs are ubiquitous in aerobic organisms that convert superoxide to H_2_O_2_ (Wang et al. 2018). PINE significantly reduced LPS-induced SOD levels, which elucidates the positive and negative effects of PINE and LPS, respectively, on ROS generation which subsequently influences SOD levels. MDA, as the final product of LPO, is a crucial marker of oxidative stress (Gou et al. 2015). In previous studies, it was investigated that taxifolin inhibit ROS production (Muramatsu et al. 2020) and quercetin glucoside has anti-oxidative effects, such as increasing SOD level and catalase activity whereas decreasing ROS and MDA levels (Dai et al. 2018). Therefore, we assumed that the active compounds in PINE, such as taxifolin or quercetin glucoside, can potentially alleviate LPS-induced oxidative stress in this study.

NO is a mediator of inflammation, and iNOS is expressed predominantly by microglia and macrophages under abnormal conditions (Kim et al. 2005). High levels of NO produced by activated macrophages may harm healthy cells (Hwang et al. 2017). The activation of macrophages by LPS stimulates iNOS expression, which induces NO production through the activity of MAP kinases and NF-κB (Hwang et al. 2017). Taxifolin and quercetin glucoside in PINE decrease NO production and the secretion of proinflammatory mediators, such as TNF-α, IL-1β and IL-6 (Lee et al. 2008; Muramatsu et al. 2020; Shi et al. 2021). Furthermore, taxifolin reduces COX-2 and PGE_2_ production (Oi et al. 2012). Quercetin glucoside decreases COX-2 and PGE_2_ production (Xu et al. 2012). In this study, PINE inhibited the LPS-induced NO overproduction and iNOS overexpression, which suggests that the anti-inflammatory effects of PINE may be mediated by taxifolin and quercetin glucoside via inhibiting iNOS overexpression and NO overproduction.

Furthermore, we quantified the levels of TNF-α, IL-1β and PGE_2_, which mediate inflammatory responses in LPS-stimulated RAW264.7 macrophages. Macrophage activation by LPS promotes the secretion of proinflammatory cytokines, such as TNF-α, IL-1β and PGE_2_ (Eliopoulos et al. 2002). PINE significantly inhibited the expression of these cytokines. Interestingly, a significant decrease in IL-1β and PGE_2_ production was observed even at low concentrations of PINE. PGE2 is an arachidonic acid metabolite produced by the enzyme COX-2 (Morteau 2000). Thus, the downregulation of COX-2 synthesis by PINE suggests that the anti-inflammatory effects of PINE are mediated by the COX-2/PGE_2_ pathway. Moreover, taxifolin and quercetin glucoside in PINE possibly downregulate the COX-2/PGE_2_ pathway because taxifolin and quercetin glucoside were known to decrease COX-2 and PGE_2_ production in previous studies (Oi et al. 2012; Xu et al. 2012; Wang et al. 2020). Based on previous and current studies about antioxidative and anti-inflammatory mechanisms, it can be inferred that taxifolin and quercetin glucoside are active compounds showing the effects of PINE. However, there are limitation since several other compounds contained in PINE, such as quinic acid, caffeoylquinic acid and taxifolin glucoside also have anti-inflammatory and antioxidant effects (Kim et al. 2010; Jang et al. 2017; Segheto et al. 2018). Thus, the comparison of the effects of each compound and PINE is needed through further research to clarify antioxidant and anti- inflammatory effects of taxifolin and quercetin glucoside in PINE as active compounds.

Animal ear oedema models are routinely used to investigate acute inflammation. Singsai et al. (2020) used a xylene-induced ear oedema mouse model to investigate the anti-inflammatory effects and antilipoxygenase activity of *Streblus asper* Lour. (Moraceae) leaf extract. Another study used a 12-*O*-tetradecanoylphorbol-13-acetate (TPA)-induced ear oedema model to measure the anti-inflammatory activity of a methanol extract of *Jefea gnaphalioides* A. Gray (Asteraceae) (Villagómez-Rodríguez et al. 2019). In the present study, we observed the anti-inflammatory effects of PINE in an arachidonic acid-induced ear oedema mouse model. Arachidonic acid exerts various physiological and pathological effects on the body, and excess arachidonic acid production can induce acute inflammatory responses, such as erythema, pyrexia and oedema (Higgins and Lees 1984). To dilute arachidonic acid, we used acetone as a solvent. Based on the preliminary our studies and other studies, the interfering effect of acetone as a solvent to dilute arachidonic acid was not shown in same or similar experiments (Ferreira et al. 2010; Mulla et al. 2010; Xu et al. 2017). To avoid the interference of vehicle and solvent used in this study, we applied the same vehicle and solvent to control group and comparison ears, respectively.

We applied 0.1, 0.3 and 1 mg of PINE to mouse ears and the concentration of the test agent was based on previous research of the topical application of 0.1, 0.3 and 1 mg extracts/ear in mouse studies (Ascari et al. 2019; Formagio-Neto et al. 2019). Dexamethasone, a synthetic glucocorticoid, induces anti-inflammatory effects through inhibition of the NF-κΒ signalling pathway (Chen et al. 2021). Dexamethasone has been commonly used as a positive control in inflammation-related studies (Huang et al. 2018). We used 0.1 mg/ear of dexamethasone for topical application based on the findings of a previous study (de Brum et al. 2016). We measured the anti-inflammatory activity of PINE in inflamed ICR mouse ears by using arachidonic acid to induce oedema in ear tissues. All tested concentrations of PINE significantly suppressed ear oedema and MPO activity. Elevated MPO levels are associated with inflammation and increased oxidative stress (Ndrepepa 2019). This study showed that MDA levels, which are a marker of oxidative stress (Gaweł et al. 2004), decreased after PINE treatment. Thus, the results suggest that PINE exerts anti-inflammatory activity not only *in vitro* but also under *in vivo* conditions.

Oxidative stress and inflammation are closely related. LPS-induced TLR activation could produce oxidative stress, and ROS could activate TLR signalling, leading to the production of inflammatory signals (Gill et al. 2010; Biswas 2016). The above-mentioned relationship induces a positive feedback process of inflammatory and oxidative stress states that can be inhibited by PINE. Although the exact step that PINE inhibits was undetermined, PINE was shown to inhibit both oxidative and inflammatory processes. PINE has a significant inhibitory effect on LPS-induced expression of proinflammatory mediators, such as iNOS and IL-6, in RAW264.7 macrophages (Venkatesan et al. 2017). This study provides both *in vitro* and *in vivo* evidence supporting the use of *P. densiflora* in folk/traditional medicine and as a dietary supplement for inflammatory conditions. However, further *in vivo* studies are needed to explore the potential application of *P. densiflora* in the clinic for the treatment of inflammatory diseases.

## Conclusions

Our finding confirmed that PINE showed antioxidant and anti-inflammatory activities in LPS-induced RAW264.7 macrophages and in an arachidonic acid-induced ICR mouse model. Moreover, LPS induced inflammatory and oxidative mediator including ROS, SOD, MDA, NO, IL-1β, TNF-α, COX-2 and PGE_2_ via mutual relation with oxidative stress and inflammation were down-regulated by PINE. Through *in vivo* study using AA induced ear oedema model, anti-inflammatory and antioxidative effects of PINE were demonstrated. According to our LC–MS analysis, several flavonoids were identified in PINE and taxifolin and quercetin glucoside among which can be active compound showing effects of PINE considering previous studies and our study. For clarifying the active compound of PINE, further study is needed to comparison with the effects of components in PINE via *in vitro* and *in vivo* study. Although several limitations in this study, our study demonstrated that PINE could be attributed to protective effect from inflammation and oxidative stress via identified several flavonoids in PINE.
